# Application of artificial intelligence in the diagnosis of subepithelial lesions using endoscopic ultrasonography: a systematic review and meta-analysis

**DOI:** 10.3389/fonc.2022.915481

**Published:** 2022-08-15

**Authors:** Xin-Yuan Liu, Wen Song, Tao Mao, Qi Zhang, Cuiping Zhang, Xiao-Yu Li

**Affiliations:** Department of Gastroenterology, The Affiliated Hospital of Qingdao University, Qingdao, China

**Keywords:** artificial intelligence, computer-assisted diagnosis, endoscopic ultrasonography, subepithelial lesions, gastrointestinal stromal tumors

## Abstract

Endoscopic ultrasonography (EUS) is the most common method for diagnosing gastrointestinal subepithelial lesions (SELs); however, it usually requires histopathological confirmation using invasive methods. Artificial intelligence (AI) algorithms have made significant progress in medical imaging diagnosis. The purpose of our research was to explore the application of AI in the diagnosis of SELs using EUS and to evaluate the diagnostic performance of AI-assisted EUS. Three databases, PubMed, EMBASE, and the Cochrane Library, were comprehensively searched for relevant literature. RevMan 5.4.1 and Stata 17.0, were used to calculate and analyze the combined sensitivity, specificity, positive likelihood ratio (PLR), negative likelihood ratio (NLR), diagnostic odds ratio (DOR), and summary receiver-operating characteristic curve (SROC). Eight studies were selected from 380 potentially relevant studies for the meta-analysis of AI-aided EUS diagnosis of SELs. The combined sensitivity, specificity, and DOR of AI-aided EUS were 0.92 (95% CI, 0.85-0.96), 0.80 (95% CI, 0.70-0.87), and 46.27 (95% CI, 19.36-110.59), respectively). The area under the curve (AUC) was 0.92 (95% CI, 0.90-0.94). The AI model in differentiating GIST from leiomyoma had a pooled AUC of 0.95, sensitivity of 0.93, specificity of 0.88, PLR of 8.04, and NLR of 0.08. The combined sensitivity, specificity, and AUC of the AI-aided EUS diagnosis in the convolutional neural network (CNN) model were 0.93, 0.81, and 0.94, respectively. AI-aided EUS diagnosis using conventional brightness mode (B-mode) EUS images had a combined sensitivity of 0.92, specificity of 0.79, and AUC of 0.92. AI-aided EUS diagnosis based on patients had a combined sensitivity, specificity, and AUC of 0.95, 0.83, and 0.96, respectively. Additionally, AI-aided EUS was superior to EUS by experts in terms of sensitivity (0.93 vs. 0.71), specificity (0.81 vs. 0.69), and AUC (0.94 vs. 0.75). In conclusion, AI-assisted EUS is a promising and reliable method for distinguishing SELs, with excellent diagnostic performance. More multicenter cohort and prospective studies are expected to be conducted to further develop AI-assisted real-time diagnostic systems and validate the superiority of AI systems.

**Systematic Review Registration:** PROSPERO (https://www.crd.york.ac.uk/PROSPERO/), identifier CRD42022303990.

## Introduction

Gastrointestinal subepithelial lesions (SELs) are tumors that originate from the muscularis mucosa, submucosa, or muscularis propria (1). According to statistics, one SEL is found in every 300 endoscopy examinations (2). SELs, including gastrointestinal stromal tumors (GIST), leiomyomas, schwannomas, neuroendocrine tumors (NET), lipomas, and ectopic pancreas, are asymptomatic and difficult to distinguish due to their similar morphology in size, shape, surface color, contour, and margin (1). GISTs are the most prevalent SELs, with a prevalence of 14–20 cases per million, and have the potential to evolve into malignancies (3, 4). Approximately 60% of patients with GISTs can be cured by surgery (5). Therefore, it is crucial to differentiate GISTs from other benign tumors.

With the development of endoscopic ultrasonography (EUS), fine-needle aspiration biopsy (FANB), immunohistochemical staining methods, and various new imaging technologies, such as contrast-enhanced harmonic EUS (CH-EUS), the approaches for diagnosing and treating SELs have improved (6). EUS as a useful tool has recently become the conventional inspection method for the discovery and diagnosis of SELs. However, the diagnostic accuracy of EUS is limited and closely related to the professional level and experience of the endoscopists (7). EUS-FNAB can be used to obtain tissue specimens for immunohistochemical staining and is the gold standard for diagnosing SELs. Nevertheless, the diagnostic yield of EUS-FNAB for SELs is not ideal, ranging from 60% to 85% (8–10). FNAB is an invasive and risky operation, and the limited sampling sites are subjectively determined by endoscopists, which may lead to missed diagnoses. Therefore, alternative methods are needed for the accurate diagnosis of SELs to avoid surgical resection of benign lesions as GISTs with malignant potential.

Recently, artificial intelligence (AI) has been extensively used in medical imaging technology, owing to its superior performance. Machine learning (ML) involves the fields of computer science and statistics, generating algorithms to analyze various types of data, and building appropriate descriptive and predictive models (11). Artificial neural networks (ANN), as mathematical models of information processing, are supervised ML models inspired by the structure of brain synaptic connections (11). A convolutional neural network (CNN) is a deep learning algorithm that shows strong performance in image recognition, classification, and processing (12). AI-aided EUS diagnostic tools have been widely applied to differentiate various types of pancreatic diseases, such as pancreatic tumors, chronic pancreatitis, and autoimmune pancreatitis (13–15). In recent years, several studies have explored the value of CNN in distinguishing SELs based on EUS images, mainly in differentiating GIST from benign lesions. In this systematic review and meta-analysis, we aimed to assess the effectiveness and accuracy of AI in diagnosing SELs using EUS images and focused on the performance of computer-aided diagnosis models in differentiating GIST from other benign lesions by comparing AI and EUS experts.

## Methods

### Search strategy

This study followed the preferred reporting items for systematic reviews and meta-analyses (PRISMA) guidelines (16). The PubMed, Embase, and Cochrane Library databases were systematically and comprehensively searched for studies on the AI-aided diagnostic accuracy of SELs under EUS with or without EUS experts as controls published until February 2022. Search terms in the title, abstract, and keywords are as follows: (“artificial intelligence” OR “AI” OR “machine learning” OR “deep learning” OR “convolutional neural network” OR “computer-assisted” OR “computer-aided” OR “neural network” OR “digital image analysis” OR “digital image processing”) AND (“endoscopic ultrasound” OR “endoscopic ultrasonography” OR “EUS”). To avoid omissions, the SELs were not included in the retrieval strategy. The retrieved articles were screened independently by two investigators (Xin-Y L and WS). Disagreements were discussed and resolved by a third researcher (TM). This protocol was registered with PROSPERO (CRD42022303990).

### Inclusion and exclusion criteria

The inclusion criteria for studies were as follows (1): prospective or retrospective study design; (2) study subjects were adult participants (≥18 years old); (3) all SELs patients were diagnosed based on histopathological diagnosis after surgical or endoscopic resection or EUS-FNAB; (4) AI algorithm was applied to the diagnosis of patients with SELs using EUS images; (5) study results demonstrated the diagnostic performance of computer-aided diagnosis (CAD) algorithms, including area under the curve (AUC), sensitivity, specificity, positive predictive value (PPV), negative predictive value (NPV), diagnostic odds ratio (DOR), or accuracy, enabling the calculation of true positive (TP), false positive (FP), true negative (TN), and false negative (FN); and (6) the manuscript was written in English. Conference proceedings, case reports, narrative and systematic reviews, meta-analyses, and studies with incomplete data (TP, FP, TN, and FN could not be calculated) were excluded. Studies with failed randomization and significant differences in baseline data between groups were also excluded from this systematic review.

### Data extraction and quality assessment

The number of histologically confirmed SELs that were true-positive (GIST considered to be GIST by AI or experts), true-negative (non-GIST considered to be non-GIST by AI or experts), false-positive (non-GIST considered to be GIST), or false-negative (GIST considered to be non-GIST) were extracted. Additionally, the first author’s name; year of publication; country where the study was conducted; study type; number of samples in the training, validation, and test sets; imaging modality; AI model; and video were also retrieved.

The Quality Assessment of Diagnostic Accuracy Studies (QUADAS-2) tool was used to evaluate the quality and potential bias of all included studies in four aspects: patient selection, index test, reference standard, and flow and timing quality (17). Regarding the problem of pre-specified thresholds, we referred to the study by Thaninee et al. and modified the problem as to whether the performance of the AI-assisted diagnostic system was validated in another cohort (18). Two reviewers (Xin-Y L and WS) independently assessed the eight studies, and conflicts were discussed and resolved with a third reviewer (TM).

### Statistical analysis

RevMan 5.4.1 (The Cochrane Collaboration, 2020, London, United Kingdom) and Stata 17.0 (StataCorp, College Station, TX, USA) were used for diagnostic meta-analysis. Published data were extracted, including the reported TP, FP, FN, TN, sensitivity, and specificity of the test datasets. The pooled sensitivity, specificity, positive likelihood ratio (PLR), negative likelihood ratio (NLR), diagnostic score, and DOR with 95% confidence intervals (CIs) were calculated and analyzed using the bivariate mixed-effects model. A summary receiver-operating characteristic curve (SROC) was also constructed, and the AUC was calculated to assess diagnostic accuracy. A funnel plot and its symmetric distribution were used to evaluate the risk of publication bias. Subgroup and meta-regression analyses were performed to explore the sources of heterogeneity. Heterogeneity among the studies was determined using I^2^ and Cochran’s Q tests. P < 0.1 generally suggests significant heterogeneity, and I^2^ >50% indicates substantial heterogeneity.

## Result

### Literature search and bias assessment

**Figure 1:**
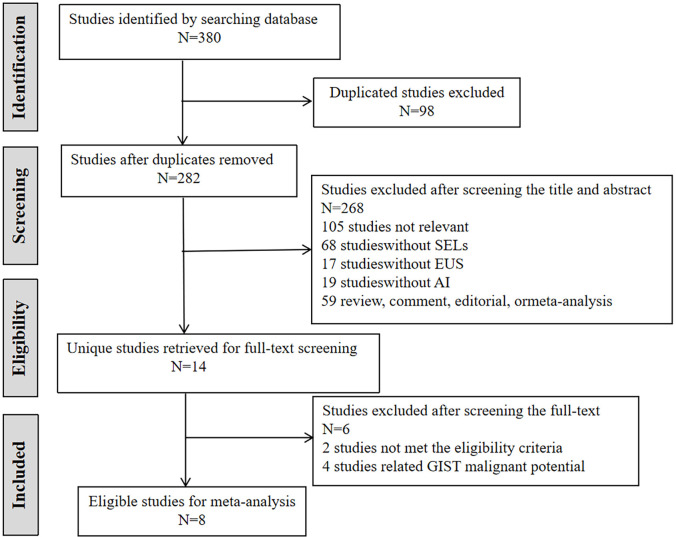
Flowchart of literature search.

The literature retrieval process and screening results are shown in **Figure 1**. Initially, 380 potentially relevant studies were retrieved from the three databases, and 98 duplicates were removed. Subsequently, 268 studies were excluded after reviewing the titles and abstracts, as they were irrelevant articles and were not suitable for the research topic or type. After screening the full text of 14 eligible studies, two studies that did not meet the eligibility criteria and four studies related to GIST malignant potential were excluded. Finally, eight studies were selected for the meta-analysis of AI-aided diagnosis of SELs according to the PRISMA flowchart (19–26).

The characteristics of all included studies are summarized in **Table 1**. A total of eight studies with 339 patients with GIST and 194 patients without GIST were included in the meta-analysis, seven of which were within the last three years. They were all retrospective studies, and one of them used both retrospective and prospective test sets in the stage of testing AI models (22). Three studies were conducted in Japan, two in South Korea, and three in China, Turkey, and the United States. Only one study developed an AI model based on contrast-enhanced harmonic EUS (CH-EUS) images, whereas the others used the conventional brightness mode (B-mode) of EUS. Considering computer-aided models, except for one study that used the ANN model, the remaining studies applied the CNN model. Only one study did not use EUS experts as controls (19). The training, validation, and testing datasets of the included studies are presented in **Supplementary Table 1**. All the studies trained and developed AI models using a large number of EUS images. One of the studies used videos from each patient divided into 0.1s intervals, yielding images to train the AI model (24).

**Table 1:** Characteristics of included studies.

Author	Year	Study type	Country	GIST	non- GIST	TP	FP	FN	TN	Reference standard	Imaging modality	AI model	EUS experts as control	Video	Reference
Vien X. Nguyen	2010	Retrospective	USA	124*/ 28	217*/ 18	100	46	24	171	Histopathology	B-mode	ANN	N	N	(19)
Yosuke Minoda	2020	Retrospective	Japan	47	13	42	4	5	9	Histopathology	B-mode	CNN	Y	N	(20)
Yoon Ho Kim	2020	Retrospective	Korea	106*/ 32	106*/ 37	88	26	18	80	Histopathology	B-mode	CNN	Y	N	(21)
Xintian Yang	2021	Retrospective & Prospective	China	30**/ 36	54**/ 41	27/32	2/14	3/4	52/27	Histopathology	B-mode	CNN	Y	N	(22)
Chang Kyo Oh	2021	Retrospective	Korea	40	14	40	2	0	12	Histopathology	B-mode	CNN	Y	N	(23)
Keiko Hirai	2021	Retrospective	Japan	85	37	84	12	1	25	Histopathology	B-mode	CNN	Y	N	(24)
Gulseren Seven	2021	Retrospective	Turkey	35	10	32	4	3	6	Histopathology	B-mode	CNN	Y	N	(25)
Hidekazu Tanaka	2022	Retrospective	Japan	42	11	38	1	4	10	Histopathology	CH-EUS	CNN	Y	Y	(26)

*ROI, region of interest, not patient.

**Data of retrospective diagnostic test.

**Figure 2:**
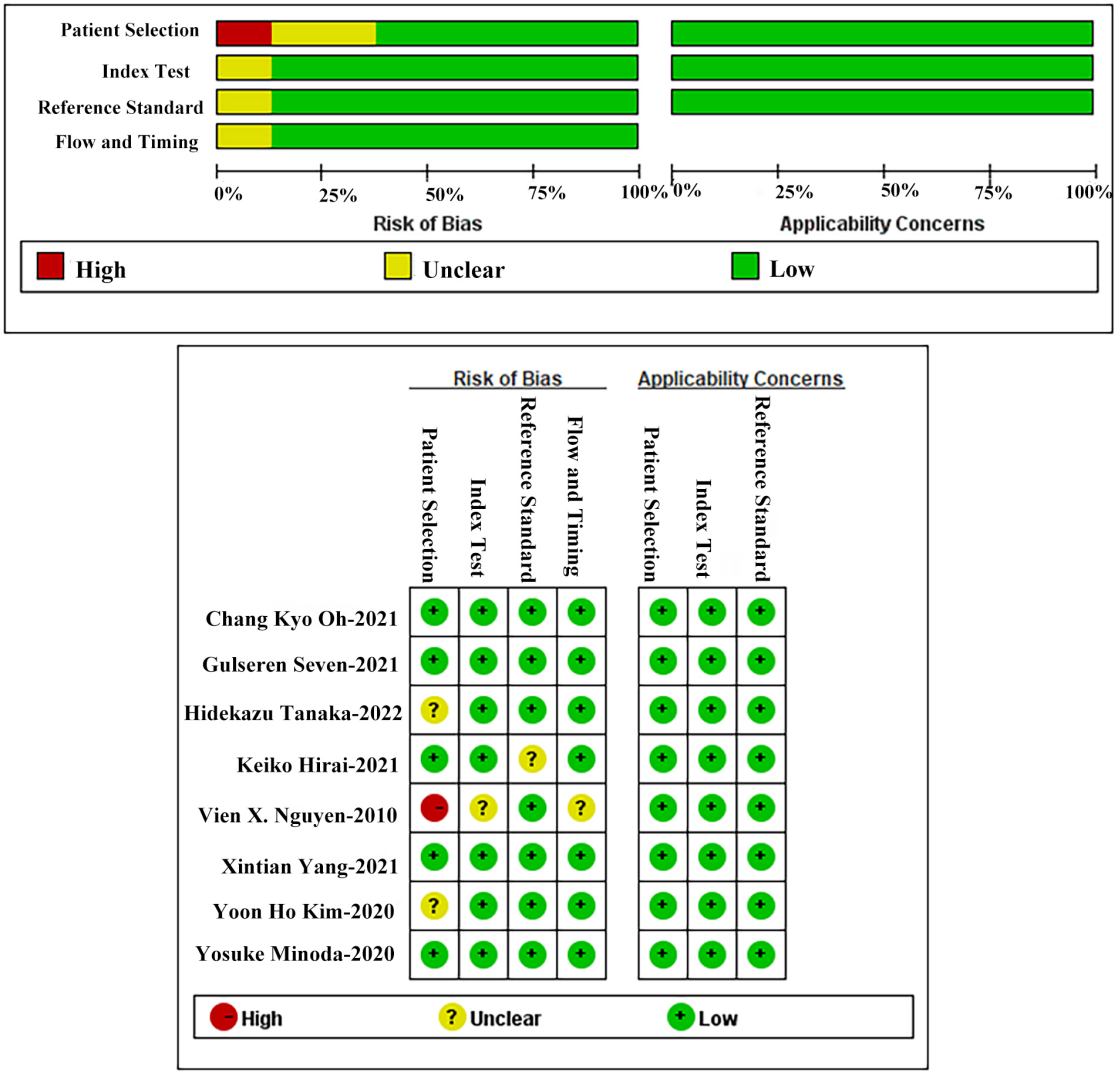
Quality assessment of included studies using QUADAS-2.

The quality and risk of bias of the included studies determined using the QUADAS-2 tool are presented in **Figure 2**. One meta-analysis of AI-aided diagnosis of GIST identified a high-risk bias in patient selection (19).

**Figure 3:**
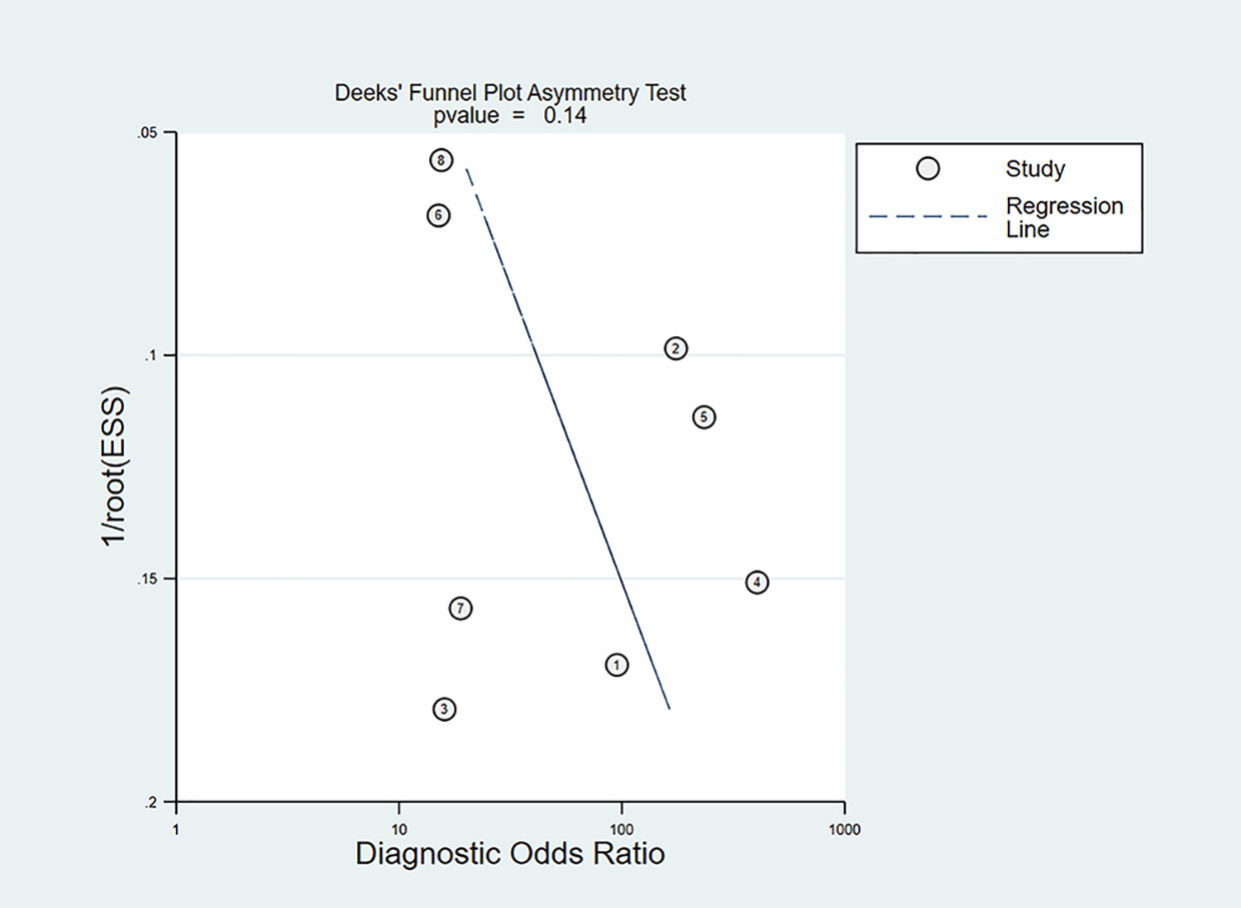
Deeks’ funnel plot of publication bias.

The slope coefficient of the Deeks’ funnel plot was symmetrical (*p* = 0.14) (**Figure 3**), indicating that publication bias was insignificant.

### Diagnostic performance of AI-assisted EUS in GIST

**Figure 4:**
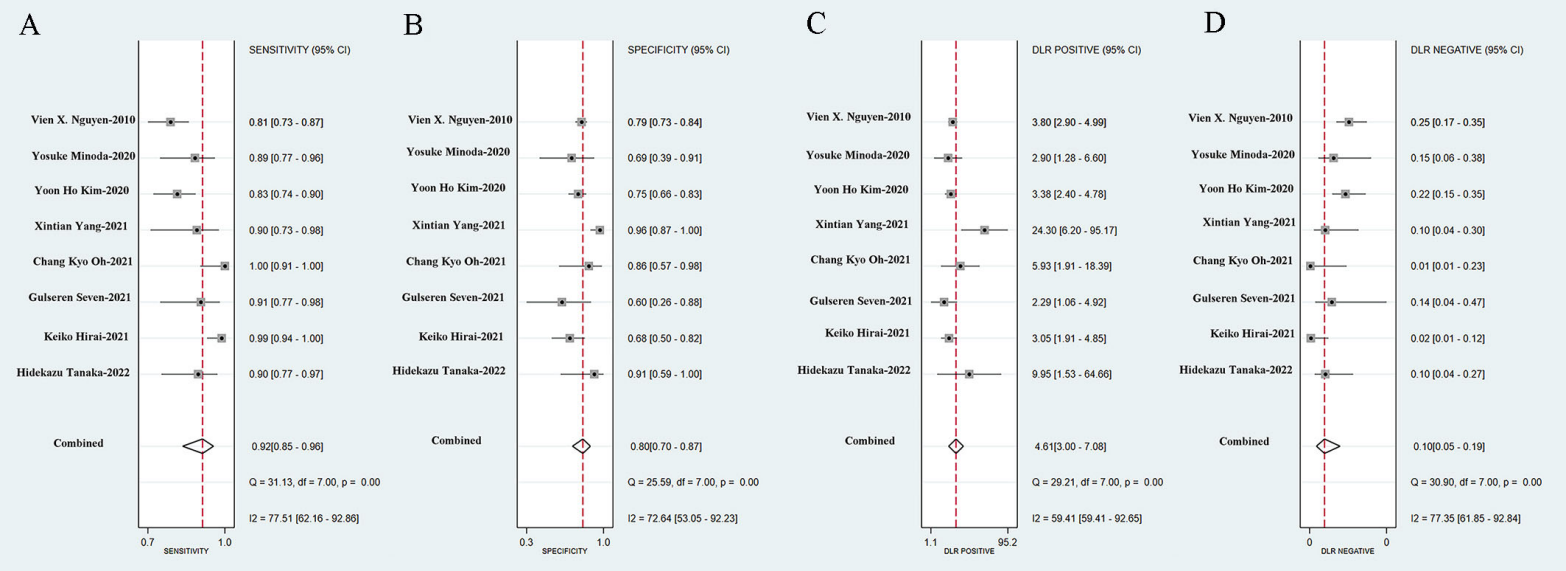
Sensitivity **(A)**, specificity **(B)**, positive likelihood ratio **(C)**, negative likelihood ratio **(D)** of AI-assisted EUS diagnosis of GIST.

**Figure 5:**
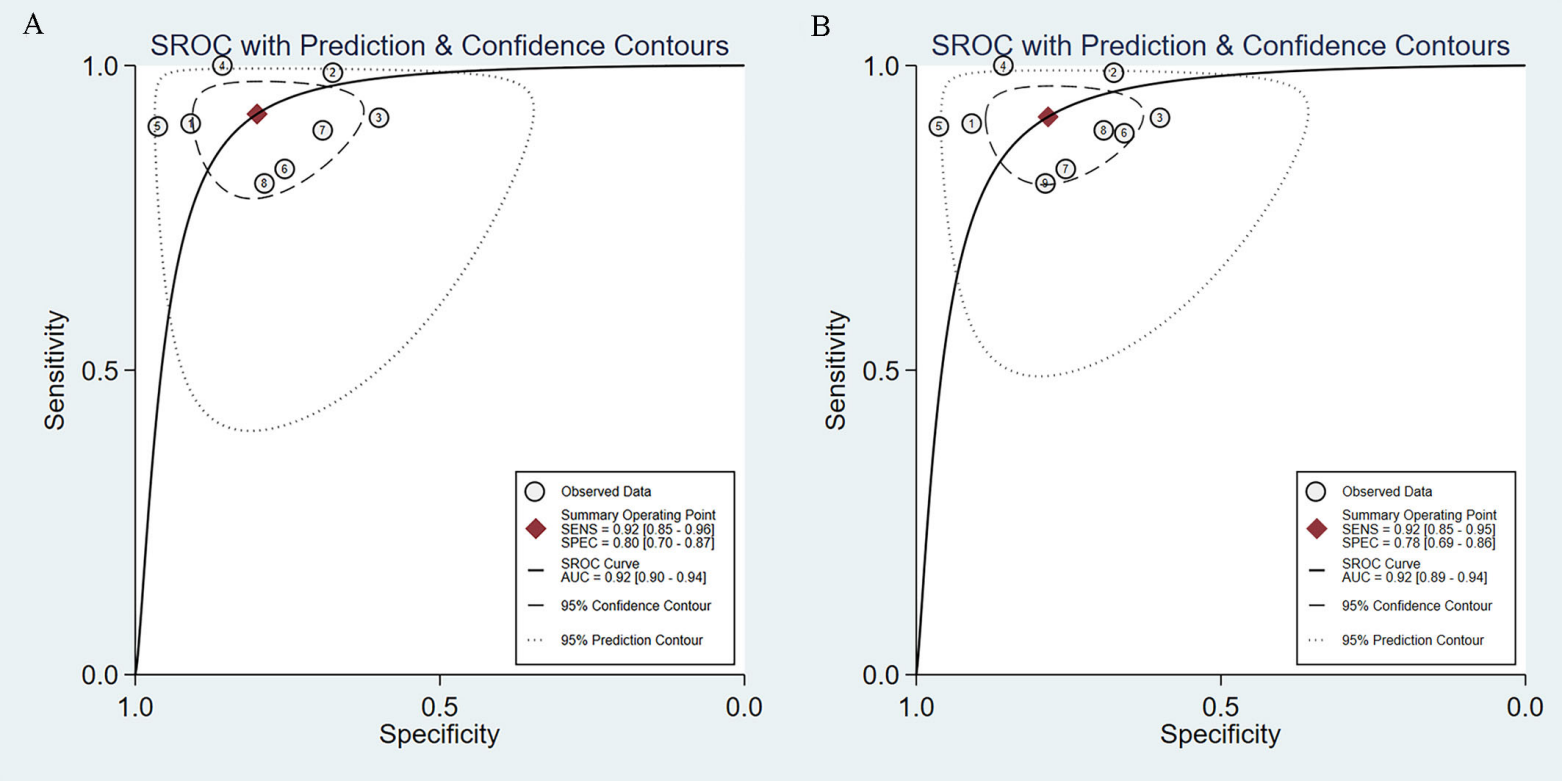
SROC curves of AI-assisted EUS diagnosis of GIST. **(A)** The SROC curve of eight studies. **(B)** SROC curve of nine datasets including prospective diagnostic test set.

We incorporated data from all retrospective diagnostic test sets and performed a meta-analysis of the eight included studies. The pooled sensitivity of AI-aided EUS diagnosis of GIST was 0.92 (95% CI, 0.85-0.96) (**Figure 4A**) and specificity was 0.80 (95% CI, 0.70-0.87) (**Figure 4B**). The pooled PLR and NLR were 4.61 (95% CI, 3.00-7.08) (**Figure 4C**) and 0.10 (95% CI, 0.05-0.19) (**Figure 4D**), respectively. The diagnostic score and DOR were 3.83 (95% CI, 2.96-4.71) and 46.27 (95% CI, 19.36-110.59), respectively (**Supplementary Figure 1**). **Figure 5A** shows the SROC curve of AI-aided EUS, with an AUC of 0.92 (95% CI, 0.90-0.94).

Subsequently, we expanded the sample size by including the data from a prospective diagnostic test set. The combined results of AI-assisted EUS diagnosis of GIST were shown as follows: AUC of 0.92 (95% CI, 0.89-0.94) (**Figure 5B**), sensitivity 0.92 (95% CI, 0.85-0.95), specificity 0.78 (95% CI, 0.69-0.86), PLR 4.23 (95% CI, 2.88-6.22), and NLR 0.11 (95% CI, 0.06-0.19) (**Supplementary Figure 2**). The diagnostic score and DOR were 3.67 (95% CI, 2.90-4.45) and 39.40 (95% CI, 18.20-85.30), respectively (**Supplementary Figure 3**).

**Figure 6:**
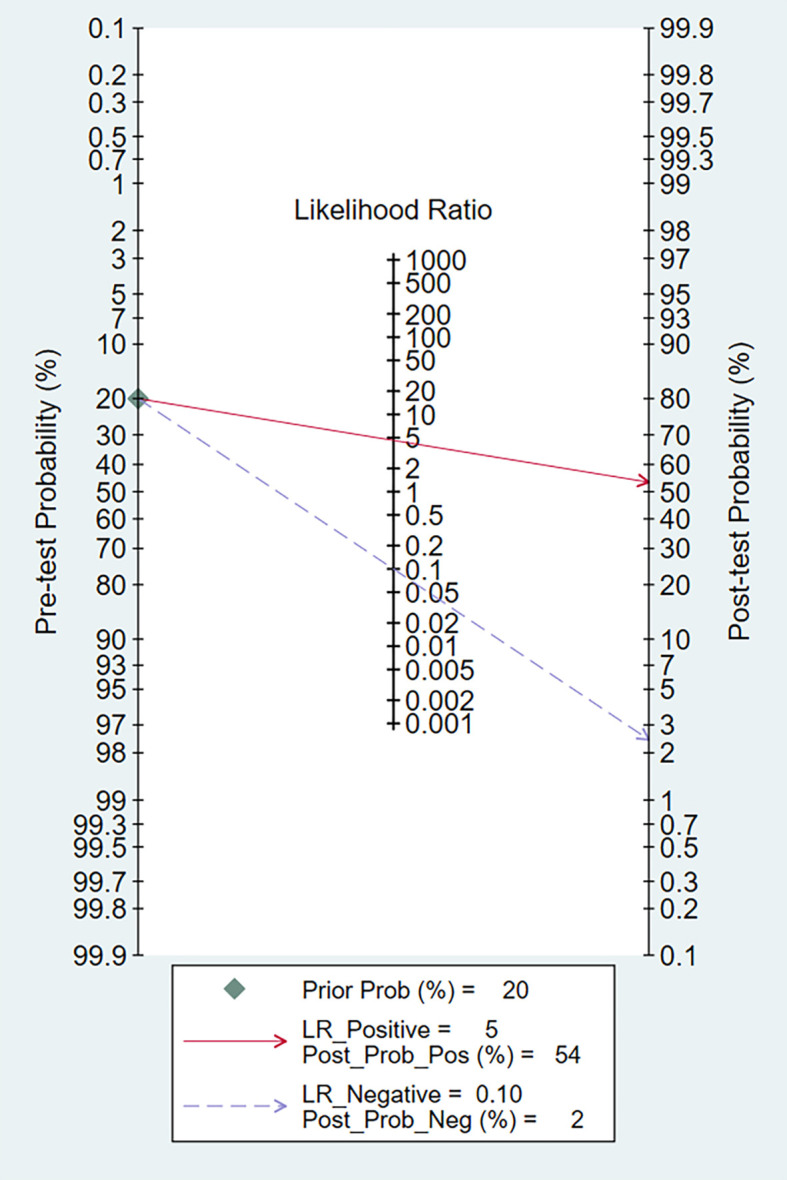
Fagan normogram for the prediction of GISTs in EUS images.

To investigate the clinical application of AI in the diagnosis of GIST, we generated a Fagan diagram (**Figure 6**). Assuming a 20% prevalence of GIST, the diagram shows a posterior probability of 54% for GIST if the test is positive, and approximately 2% for a negative test.

### Subgroup analysis of AI-assisted EUS

The specific types of SELs in the included studies are shown in **Supplementary Table 2**. One study involved five SELs, including GIST, leiomyomas, schwannomas, NET, and ectopic pancreas (24). Four studies developed AI only for the differential diagnosis of GIST and leiomyoma (22, 23, 25, 26), and a subgroup analysis of these four studies was conducted to explore the discriminating ability of the two diseases. The AI model had a pooled AUC of 0.95 (95% CI, 0.93-0.97), sensitivity of 0.93 (95% CI, 0.87-0.97), specificity of 0.88 (95% CI, 0.71-0.96), PLR of 8.04 (95% CI, 2.92-22.18), and NLR of 0.08 (95% CI, 0.04-0.15) (**Supplementary Figures 4**, **5**).

**Figure 7:**
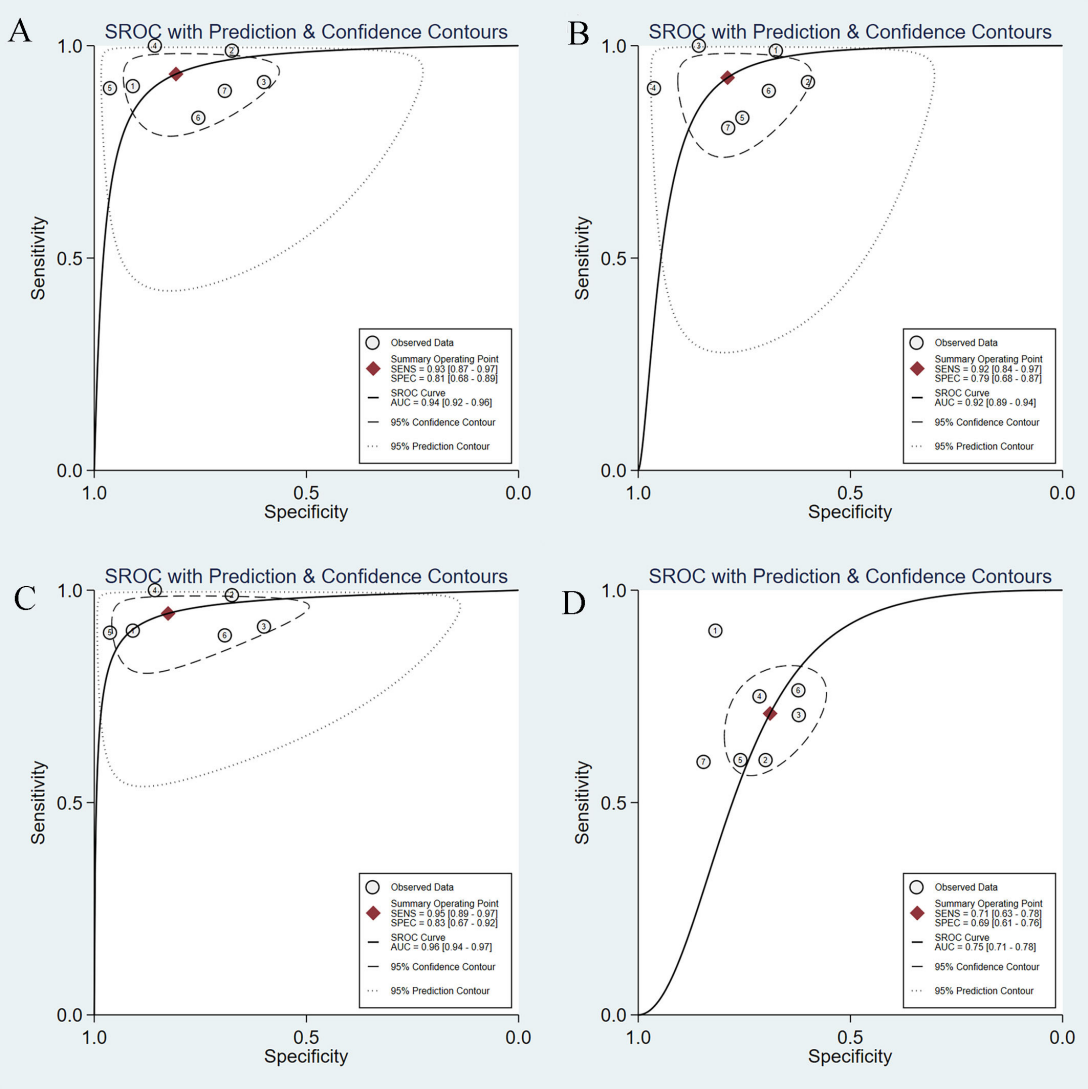
SROC curves of AI-assisted EUS and EUS experts in diagnosis of GIST. **(A)** SROC curve of seven studies on CNN AI-models. **(B)** SROC curve of seven studies on imaging modality. **(C)** SROC curve of seven studies based on patients. **(D)**The SROC curve of the EUS experts.

We performed a subgroup analysis after excluding Nguyen’s study, as the AI model adopted was ANN. The combined sensitivity and specificity of AI-assisted EUS diagnosis of GIST on the CNN model were 0.93 (95% CI, 0.87-0.97) and 0.81 (95% CI, 0.68-0.89) (**Supplementary Figures 6A, B**), respectively. The pooled PLR was 4.85 (95% CI, 2.81-8.36) and NLR was 0.08 (95% CI, 0.04-0.17) (**Supplementary Figures 6C, D**). **Figure 7A** shows the SROC curve of the AI-assisted EUS, with an AUC of 0.94 (95% CI, 0.92-0.96). The I^2^ was 50.57% for PLR, 74.05% for sensitivity, 71.16% for specificity, and 73.61 for NLR, indicating that significant heterogeneity existed in the pooled sensitivity, specificity, and NLR.

We also conducted a subgroup analysis of seven studies on imaging modalities without CH-EUS. The AI model had a pooled AUC of 0.92 (95% CI, 0.89-0.94) (**Figure 7B**), sensitivity of 0.92 (95% CI, 0.84-0.97), specificity of 0.79 (95% CI, 0.68-0.87), PLR of 4.39 (95% CI, 2.85-6.78), and NLR of 0.10 (95% CI, 0.04-0.21) (**Supplementary Figure 7**). However, the heterogeneity within the subgroups was still significantly high.

A subgroup analysis of AI-assisted EUS diagnosis of GIST was performed on the study subjects, namely six studies based on patients and not regions of interest. As shown in **Supplementary Figure 8**, the combined sensitivity, specificity, PLR, and NLR were 0.95 (95% CI, 0.89-0.97), 0.83 (95% CI, 0.67-0.92), 5.43 (95% CI, 2.75-10.71), 0.07 (95% CI, 0.03-0.13), respectively. The SROC curve, with an AUC of 0.96 (95% CI, 0.94-0.97), is displayed in **Figure 7C**. I^2^ was 38.69% for PLR, 51.31% for NLR, 58.46% for sensitivity, and 71.06% for specificity, indicating a low degree of heterogeneity in PLR, whereas there was moderate heterogeneity in NLR, sensitivity, and specificity.

**Figure 8:**
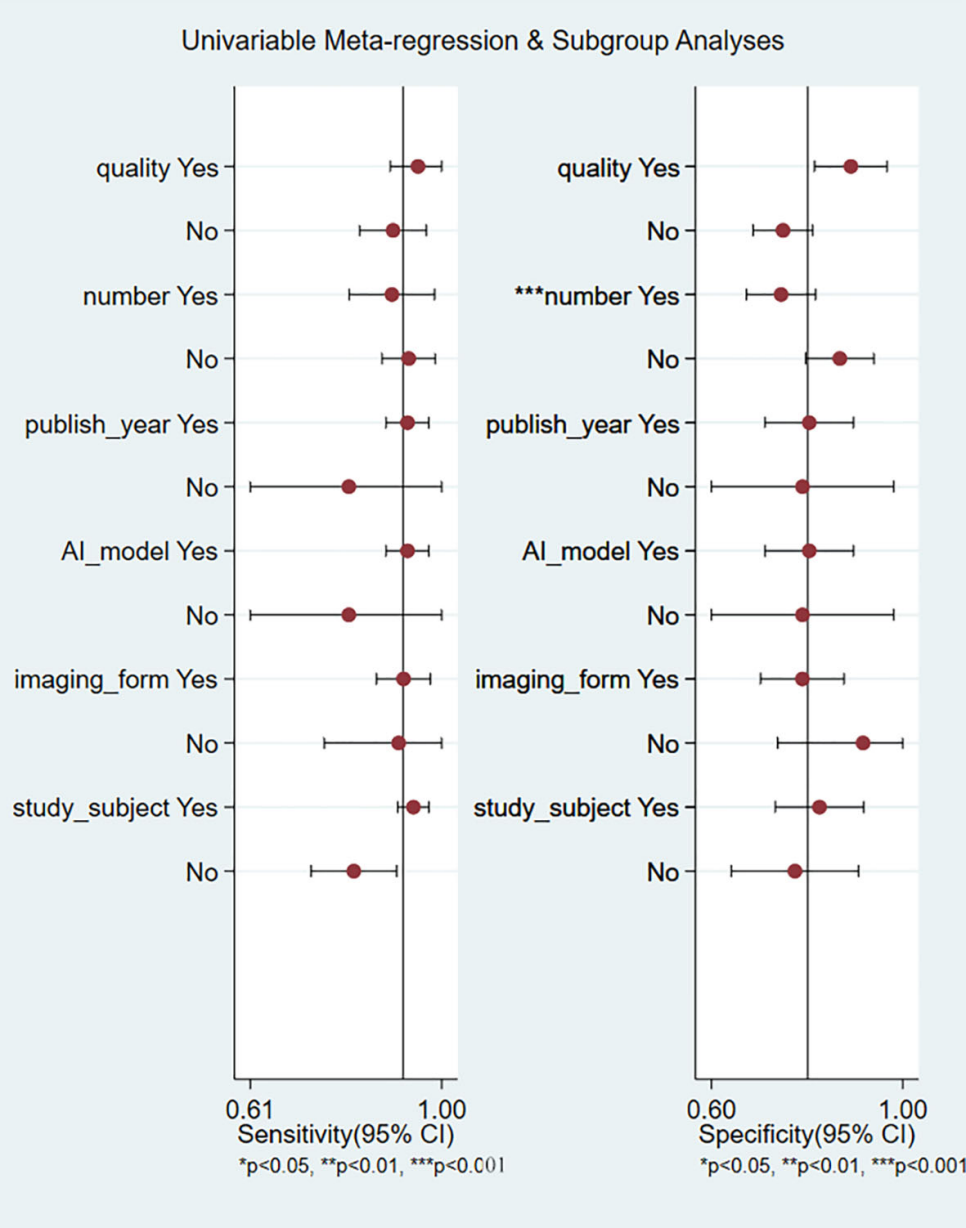
Univariate meta-regression for the reason of heterogeneity in sensitivity and specificity.

To further explore the source of heterogeneity, we performed meta-regression analysis. The number of samples was a major source of heterogeneity in univariate meta-regression analysis (*p <*0.001, **Figure 8**). Study quality (*p* = 0.03) and study subjects (*p* = 0.01) were major sources of heterogeneity in the joint meta-regression model (**Table 2**).

**Table 2:** P-value of parameters in the joint model.

Parameter	LRTChi2	P-value	I2	I2lo	I2hi
Quality*	6.73	0.03	70	34	100
Number	4.59	0.10	56	2	100
Publish year	2.11	0.35	5	0	100
AI model	2.11	0.35	5	0	100
Imaging form	0.90	0.64	0	0	100
Study subject*	8.85	0.01	77	51	100

*p <0.05

### Comparison between AI and EUS experts

**Figure 9:**
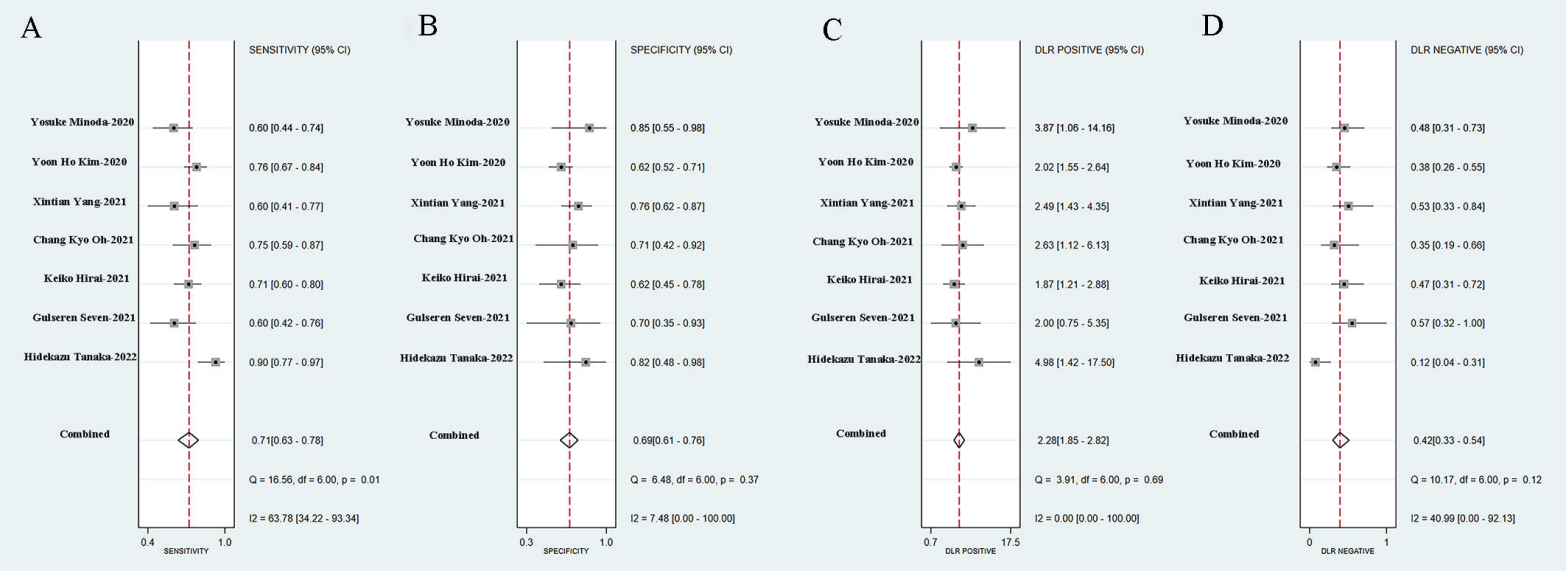
Sensitivity **(A)**, specificity **(B)**, positive likelihood ratio **(C)**, negative likelihood ratio **(D)** of EUS experts in diagnosis of GIST.

Seven studies simultaneously tested the accuracy of EUS experts in the diagnosis of GIST. All EUS experts performed more than 500 EUS examinations or had at least 5-year experience in evaluating gastrointestinal SELs. The SROC curve of the EUS experts, with an AUC of 0.75 (95% CI, 0.71-0.78), is displayed in **Figure 7D**. The pooled sensitivity of EUS experts in diagnosing GIST was 0.71 (95% CI, 0.63-0.78) (**Figure 9A**) and specificity was 0.69 (95% CI, 0.61-0.76) (**Figure 9B**). The combined PLR and NLR are 2.28 (95% CI, 1.85-2.82) (**Figure 9C**) and 0.42 (95% CI, 0.33-0.54) (**Figure 9D**), respectively. There was little heterogeneity in the specificity (*p* = 0.37), PLR (*p* = 0.69), and NLR (*p* = 0.12).

**Figure 10:**
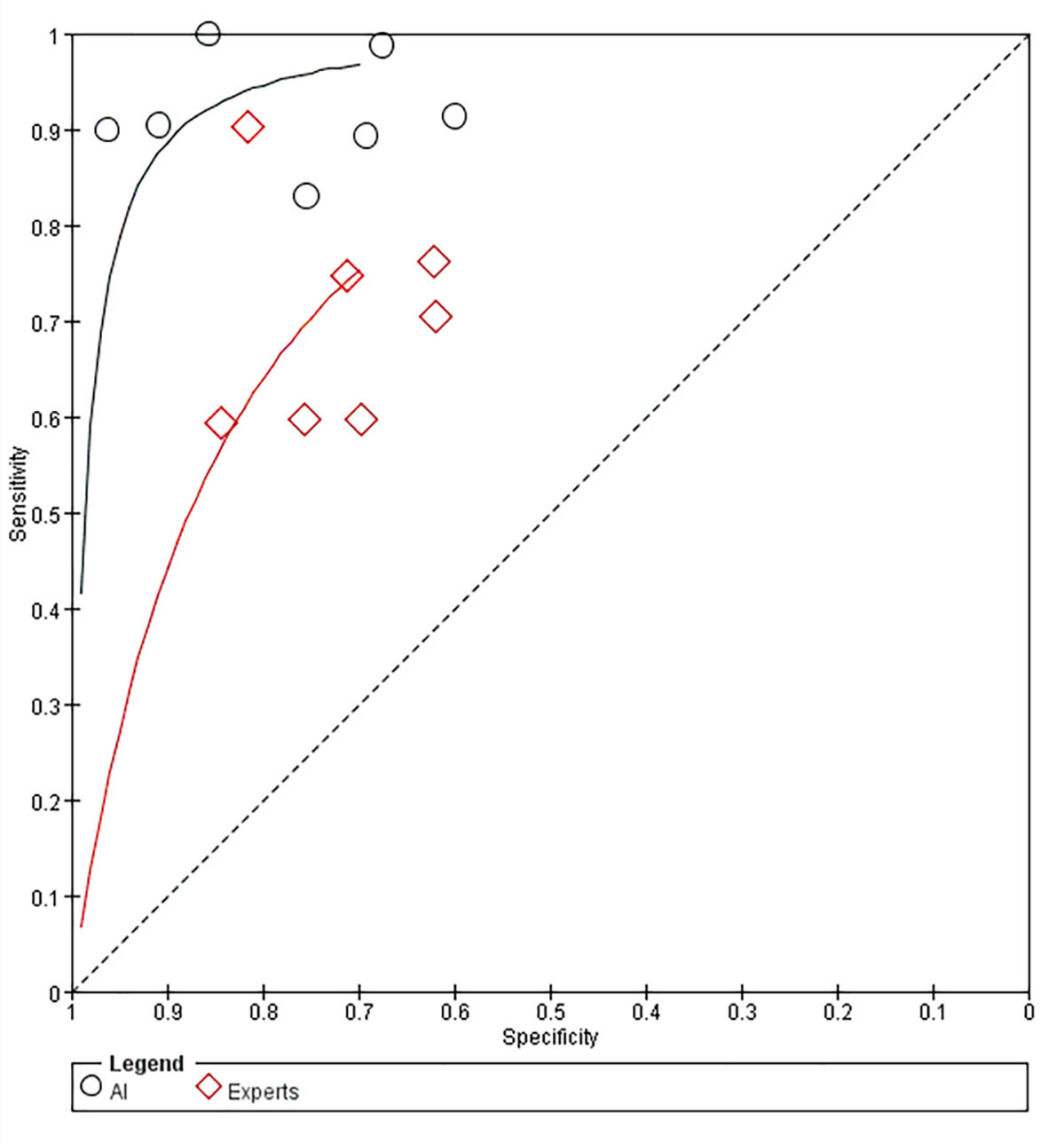
Comparison of SROC curves between AI-assisted EUS models and EUS experts.

For diagnosis of GIST under EUS, AI was superior to EUS experts in terms of sensitivity [0.93 (95% CI, 0.87-0.97) vs. 0.71 (95% CI, 0.63-0.78)], specificity [0.81 (95% CI, 0.68-0.89) vs. 0.69 (95% CI, 0.61-0.76)], and PLR [4.85 (95% CI, 2.81-8.36) vs. 2.28 (95% CI, 1.85-2.82)], and NLR [0.08 (95% CI, 0.04-0.17) vs. 0.42 (95% CI, 0.33-0.54)]. **Figure 10** shows the comparison of SROC curves between AI-assisted EUS models and EUS experts with AUC of 0.94 (95% CI, 0.92-0.96) vs. 0.75 (95% CI, 0.71-0.78), suggesting that AI-assisted EUS models have better diagnostic performance.

## Discussion

With the application of artificial intelligence in medical imaging technology, an increasing number of diseases have advanced their diagnosis and treatment methods. In this systematic review and meta-analysis, we explored the application of computer-aided diagnosis systems in gastrointestinal SELs and found that artificial intelligence algorithm models have excellent diagnostic performance with a sensitivity of 0.92 (95% CI, 0.85-0.96) and specificity of 0.80 (95% CI, 0.70-0.87). EUS is currently the most accurate and prevalent imaging modality for evaluating gastrointestinal SELs because of its ability to penetrate tissue layers and, thus, most likely identify the origin of the lesion (1). A previous study has shown that CH-EUS has better diagnostic performance than B-mode EUS in distinguishing leiomyomas from GIST and discriminating the risk stratification of GIST (27). In addition to improving the equipment performance and imaging technology of EUS, the application of artificial intelligence undoubtedly compensates for the limitations of EUS. With the help of the AI system, it is expected to shorten the diagnostic time, improve diagnostic efficiency, and reduce the misdiagnosis rate of GIST and other benign lesions, thus avoiding unnecessary EUS tests, invasive biopsies, and surgical operations.

In our initial literature search, we found that Kim and Lee used digital image analysis of objective information provided by EUS images to diagnose gastric stromal tumors (28, 29). We excluded these two studies because they were limited to analyzing the features of EUS images and did not develop corresponding AI models. We also found four studies that explored the application of AI in the malignancy stratification of GISTs, and the overall accuracy of the AI models in predicting the malignant potential of GISTs was 66.0%-83.4% (30–33). During the literature search, we found that several studies have explored the application of AI in SELs, especially GISTs. Therefore, we systematically and comprehensively summarized the application of AI-assisted EUS for the diagnosis of SELs. Although there are many types of SELs, most studies classified SELs into two categories: GIST and non-GIST, to explore the accuracy of AI-assisted EUS. In four studies, the non-GISTs only referred to leiomyoma, and we performed a subgroup analysis (22, 23, 25, 26). Nguyen et al. developed an ANN with excellent performance for differentiating lipomas (AUC=0.92), carcinoids (AUC=0.86), and GISTs (AUC=0.89) (19). Despite the SELs involved in the Minoda’s research, including GIST, leiomyoma, schwannoma, and aberrant pancreas, the results section was still divided into GIST and non-GIST for exploration (20). Kim et al. utilized CNN-CAD to first classify SELs into GIST and non-GIST tumors, and then sub-classified the non-GIST tumors into leiomyomas and schwannomas. Accuracy of the CNN-CAD system in differentiating leiomyomas from schwannomas was 85.0% (95% CI: 81.6-87.7%) (21). In the Hirai’s study, accuracy of the AI system for five-category classification was 86.1%, including GIST, leiomyoma, schwannoma, NET, and ectopic pancreas (24).

Nguyen trained, constructed, and internally validated an ANN through unsupervised and supervised learning based on the features extracted through texture analysis (19). In the traditional sense, ANN is a type of machine learning (ML). As a computer application, ML can recognize patterns in training data and generate mathematical models to develop an AI system to realize the recognition and prediction function, similar to the learning behavior of humans (13). Other studies trained CNN models using deep-learning algorithms. Deep learning-based analysis does not need to measure characteristic values, as they can be automatically and accurately identified, thereby demonstrating greater diagnostic ability (34). This is consistent with our findings that the combined AUC of CNN model after excluding the ANN model was improved from 0.92 (95% CI, 0.90-0.94) to 0.94 (95% CI, 0.92-0.96).

Heterogeneity is a prominent issue in this meta-analysis. Although we performed subgroup analyses based on the AI models, imaging modalities, and study subjects, the heterogeneity was not completely eliminated. Possible reasons for this are as follows: First, we have to consider the diversity of clinical samples, as most of the included studies were from different countries, and the manufacturers and models of EUS were inconsistent. In addition, the sample size was not sufficiently large. Second, methodological diversity should be considered. The specific algorithms, tools used, and parameter settings were not uniform, despite the fact that seven studies applied the CNN deep-learning model. The EUS expert group had little heterogeneity, probably because all EUS experts were selected on the basis of having performed more than 500 EUS examinations or having at least 5 years of experience in assessing gastrointestinal SELs. Additionally, different trial designs also contributed to the heterogeneity. Only two studies applied training, validation, and test sets (22, 24). Others merely had two datasets: one set to develop the AI model and the other to validate it. Considering the existence of heterogeneity, we avoided directly adopting a fixed-effects model.

In this review and meta-analysis, the diagnostic performance of AI models was superior to EUS experts, with the accuracy of 0.94 (95% CI, 0.92-0.96) vs. 0.75 (95% CI, 0.71-0.78). Additionally, two studies also investigated the diagnostic accuracy of AI-assisted EUS according to the size of SELs, ≥ 20 mm and <20 mm. Minoda et al. found that the accuracy, sensitivity, and specificity of SELs ≥ 20 mm between AI-assisted EUS and EUS experts were 90.0% vs. 53.3%, 91.7% vs. 50.0%, and 83.3% vs. 83.3%, respectively. The diagnostic performance for SELs ≥ 20 mm of AI-assisted EUS was significantly better than that of EUS experts, with an AUC of 0.965 vs. 0.684 (*p* = 0.007) (20). Tanaka et al. discovered that the diagnostic performance of AI and experts was completely consistent for cases with lesions <20 mm, but the specificity and accuracy of AI in diagnosing GISTs ≥ 20 mm were superior to those of experts (87.5% vs. 75.0% and 88.9% vs. 86.1%, respectively) (26). Therefore, we need to further develop and improve artificial intelligence algorithms to improve their performance in the diagnosis of small lesions.

This is the first systematic review and meta-analysis of AI-assisted EUS for SEL diagnosis. We summarized recent advances in AI in the diagnosis and differential diagnosis of SELs and evaluated the overall diagnostic performance of AI. Our meta-analysis also has some limitations. Although no publication bias existed, the number of eligible studies was limited (n=8) and most of the included studies were retrospective. Future studies are expected to expand the sample size, supplement videos, add external validation datasets, and conduct prospective real-time clinical studies to further confirm the credibility of AI diagnostic performance. In addition, the issue of heterogeneity among studies is also discussed above.

In conclusion, AI-assisted EUS is a promising and reliable method for differentiating SELs with high accuracy, and may become an important tool to assist endoscopists in diagnosing SELs in the near future.

## Data availability statement

The original contributions presented in the study are included in the article/**Supplementary Material**. Further inquiries can be directed to the corresponding author.

## Author contributions

X-YLiu, WS, and TM was responsible for literature search and screening, data extraction, analysis and interpretation, and manuscript writing. QZ and CZ was responsible for the statistical analysis and revising the manuscript. X-YLi was responsible for revising the manuscript, financial support, and final approval of the manuscript. All authors have read and approved the final version of the manuscript.

## Funding

The study was supported by the National Natural Science Foundation (No. 81802777), the “Clinical medicine + X” scientific research project of Affiliated Hospital of Qingdao University, and Qingdao Chinese Medicine Technology Project (2021-zyym26).

## Acknowledgments

We would like to thank Editage (www.editage.com) for English language editing. And we thank all the authors for helping with the writing and publication of this article.

## Conflict of interest

The authors declare that the research was conducted in the absence of any commercial or financial relationships that could be construed as a potential conflict of interest.

## Publisher’s note

All claims expressed in this article are solely those of the authors and do not necessarily represent those of their affiliated organizations, or those of the publisher, the editors and the reviewers. Any product that may be evaluated in this article, or claim that may be made by its manufacturer, is not guaranteed or endorsed by the publisher.

## Supplementary material

Supplementary figure 1 The diagnostic score and DOR of AI-assisted EUS diagnosis of GIST.

Supplementary figure 2 Sensitivity **(A)**, specificity **(B)**, positive likelihood ratio **(C)**, negative likelihood ratio **(D)** of AI-assisted EUS diagnosis of GIST including prospective test set.

Supplementary figure 3 The diagnostic score and DOR of AI-assisted EUS diagnosis of GIST including prospective diagnostic test set.

Supplementary figure 4 SROC curves of AI-assisted EUS of four studies for the diagnosis of GIST and leiomyoma.

Supplementary figure 5 Sensitivity **(A)**, specificity **(B)**, positive likelihood ratio **(C)**, negative likelihood ratio **(D)** of AI-assisted EUS for the differential diagnosis of GIST and leiomyoma.

Supplementary figure 6 Sensitivity **(A)**, specificity **(B)**, positive likelihood ratio **(C)**, negative likelihood ratio **(D)** of AI-assisted EUS of seven studies on CNN AI-models.

Supplementary figure 7 Sensitivity **(A)**, specificity **(B)**, positive likelihood ratio **(C)**, negative likelihood ratio **(D)** of AI-assisted EUS of seven studies on imaging modality.

Supplementary figure 8 Sensitivity **(A)**, specificity **(B)**, positive likelihood ratio **(C)**, negative likelihood ratio **(D)** of AI-assisted EUS of seven studies based on patients.

Supplementary Table 1 The training, validation, and test datasets of included studies.

Supplementary Table 2 Types of SELs of included studies.
