# Supplementary figures and images for: Application of artificial intelligence in the diagnosis of subepithelial lesions using endoscopic ultrasonography: a systematic review and meta-analysis
Source: Front Oncol. 2022 Aug 15;12:915481. doi: 10.3389/fonc.2022.915481 (PMC9420906; doi:10.3389/fonc.2022.915481)

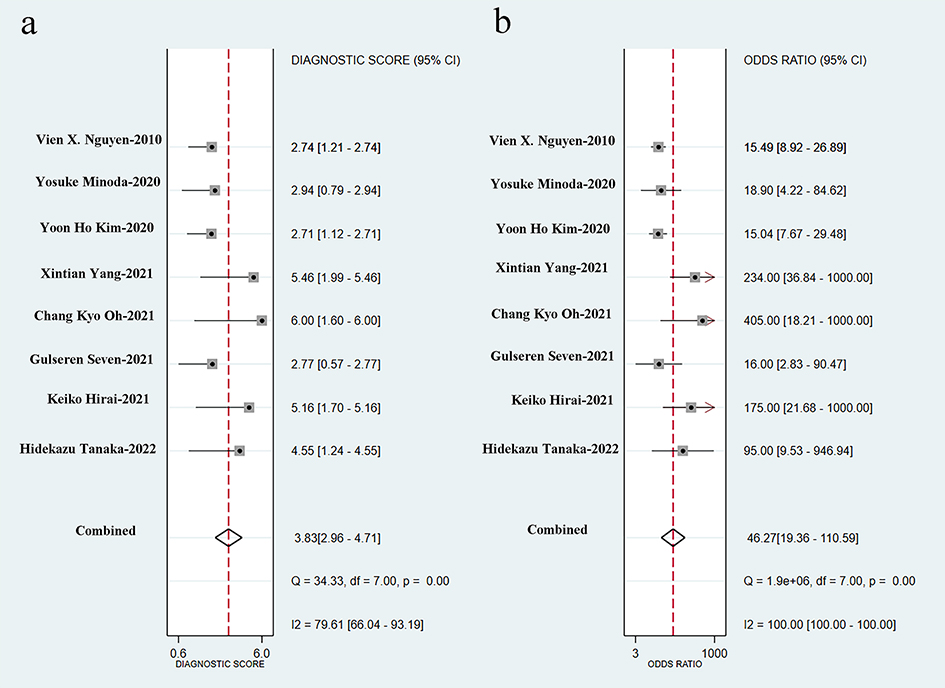

Supplement: Supplementary figure 1 — The diagnostic score and DOR of AI-assisted EUS diagnosis of GIST. [file Image_1.jpeg]

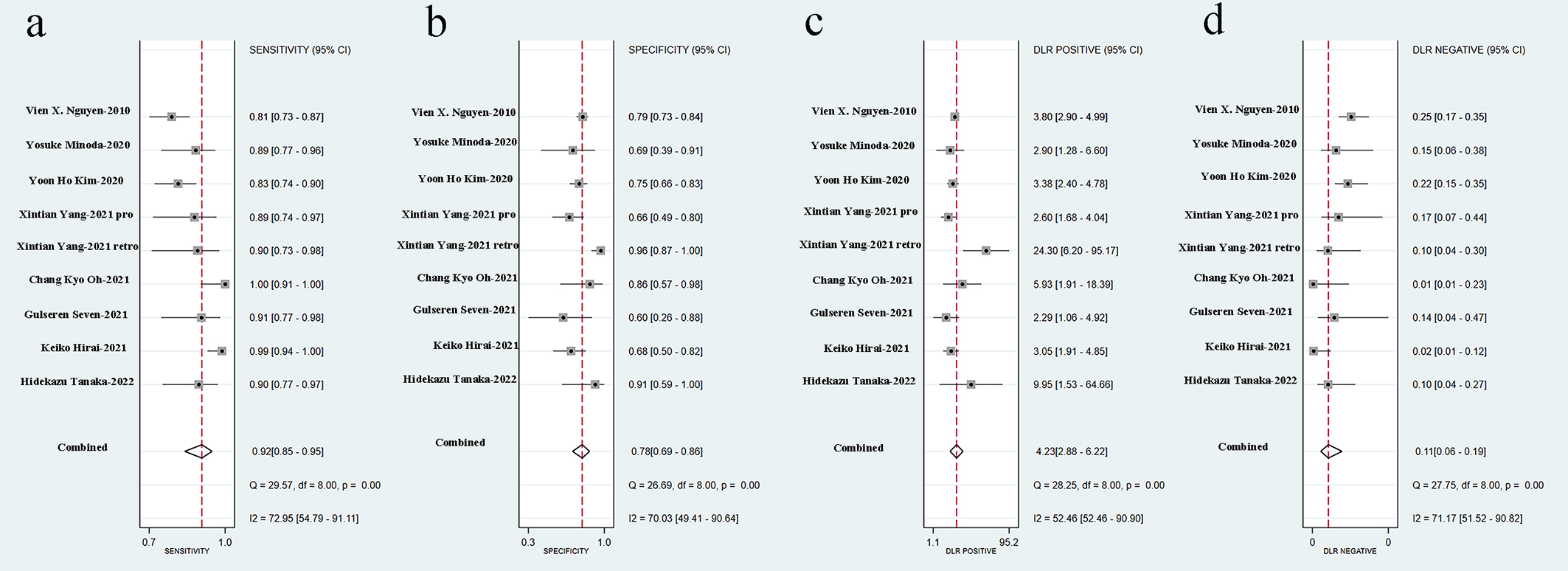

Supplement: Supplementary figure 2 — Sensitivity (A), specificity (B), positive likelihood ratio (C), negative likelihood ratio (D) of AI-assisted EUS diagnosis of GIST including prospective test set. [file Image_2.jpeg]

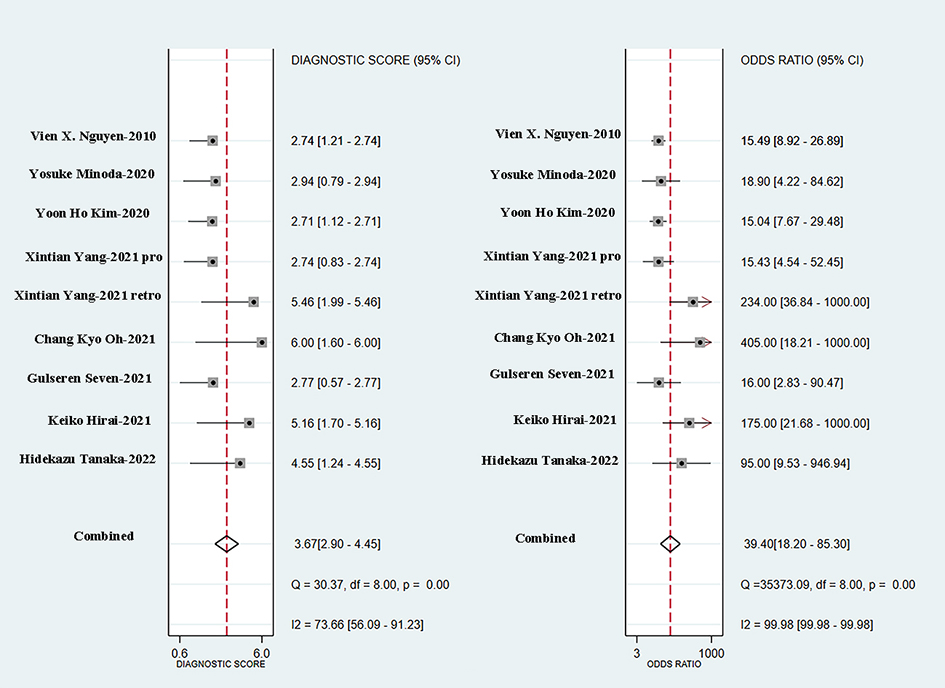

Supplement: Supplementary figure 3 — The diagnostic score and DOR of AI-assisted EUS diagnosis of GIST including prospective diagnostic test set. [file Image_3.jpeg]

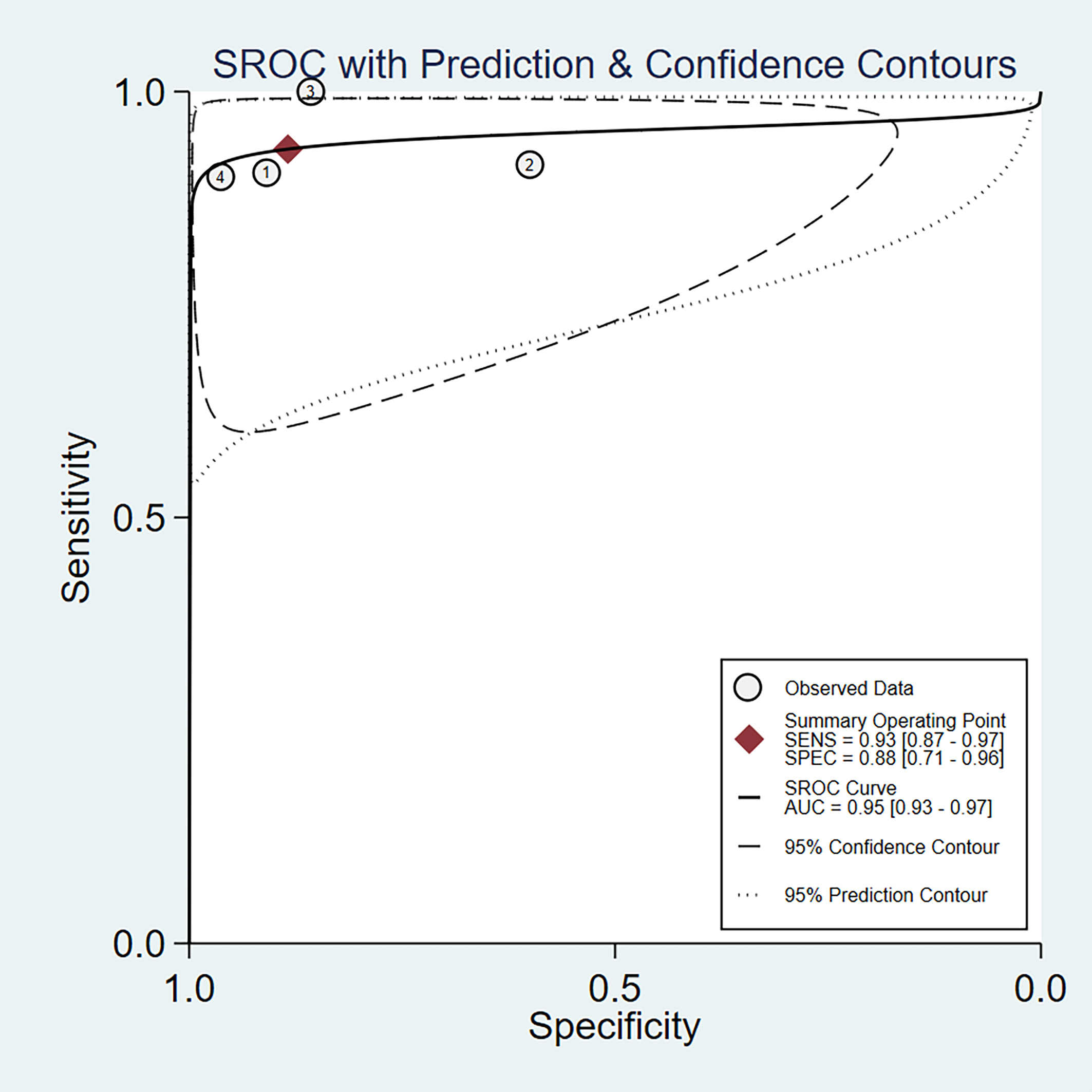

Supplement: Supplementary figure 4 — SROC curves of AI-assisted EUS of four studies for the diagnosis of GIST and leiomyoma. [file Image_4.jpeg]

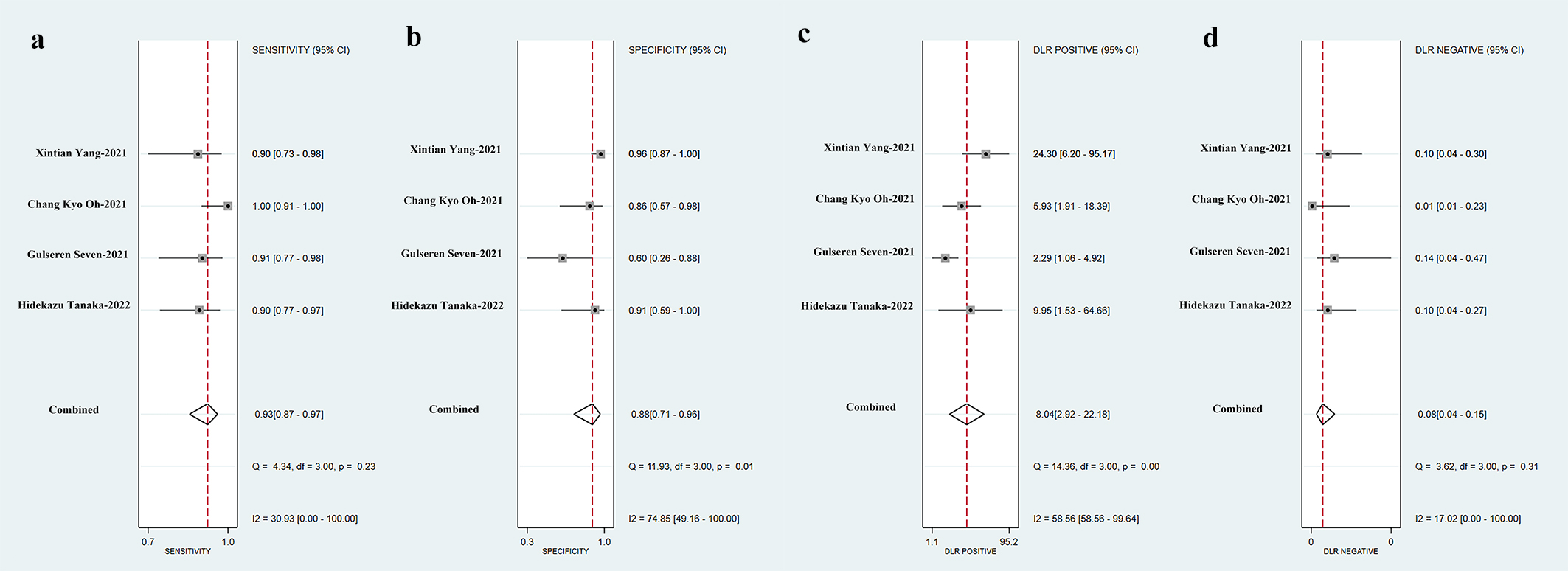

Supplement: Supplementary figure 5 — Sensitivity (A), specificity (B), positive likelihood ratio (C), negative likelihood ratio (D) of AI-assisted EUS for the differential diagnosis of GIST and leiomyoma. [file Image_5.jpeg]

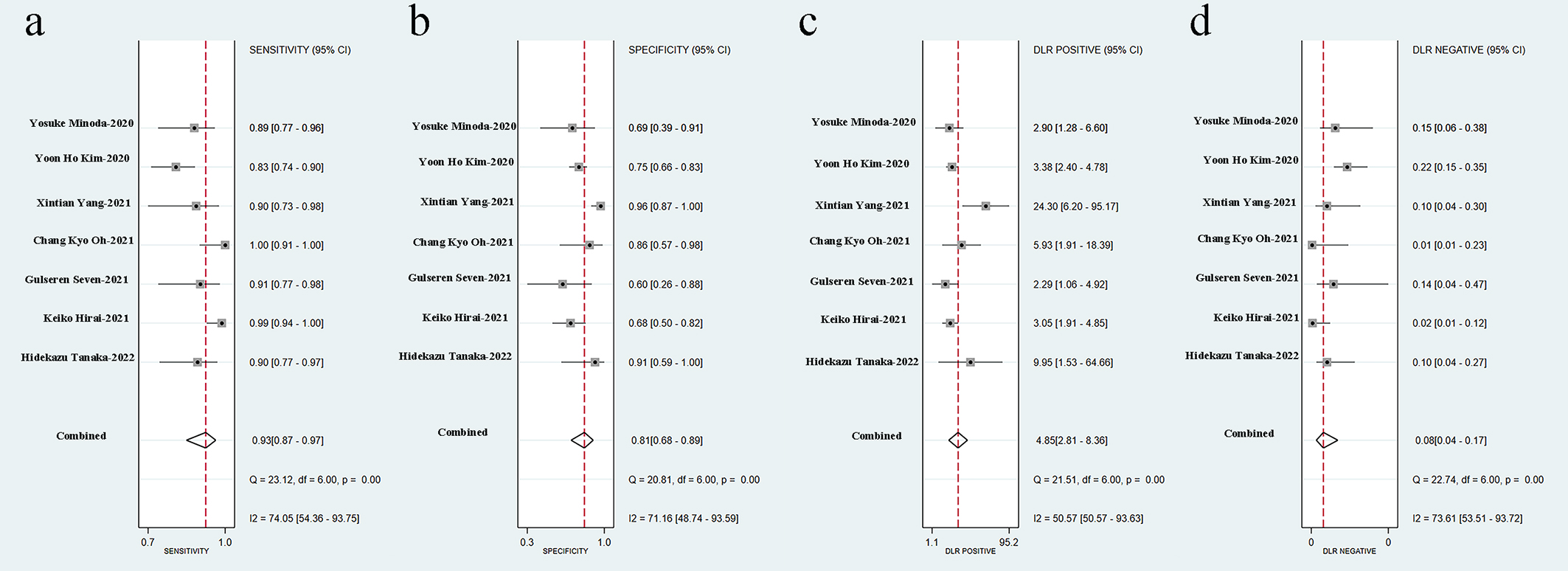

Supplement: Supplementary figure 6 — Sensitivity (A), specificity (B), positive likelihood ratio (C), negative likelihood ratio (D) of AI-assisted EUS of seven studies on CNN AI-models. [file Image_6.jpeg]

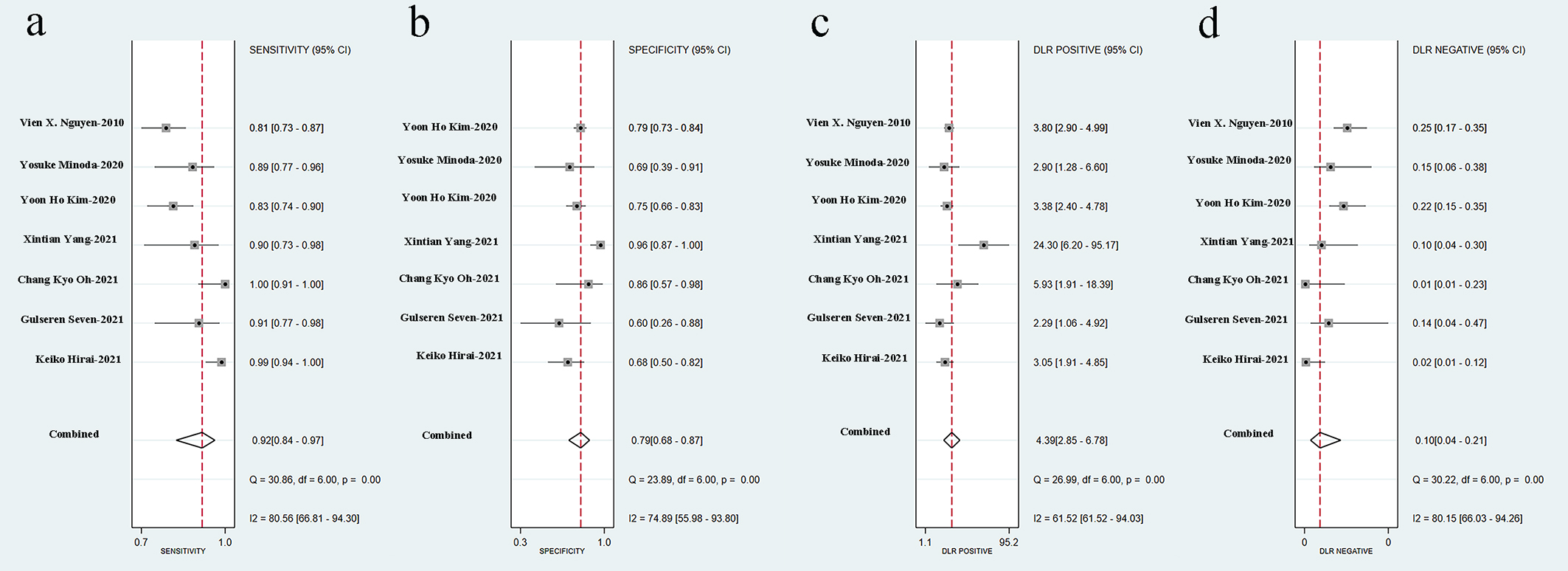

Supplement: Supplementary figure 7 — Sensitivity (A), specificity (B), positive likelihood ratio (C), negative likelihood ratio (D) of AI-assisted EUS of seven studies on imaging modality. [file Image_7.jpeg]

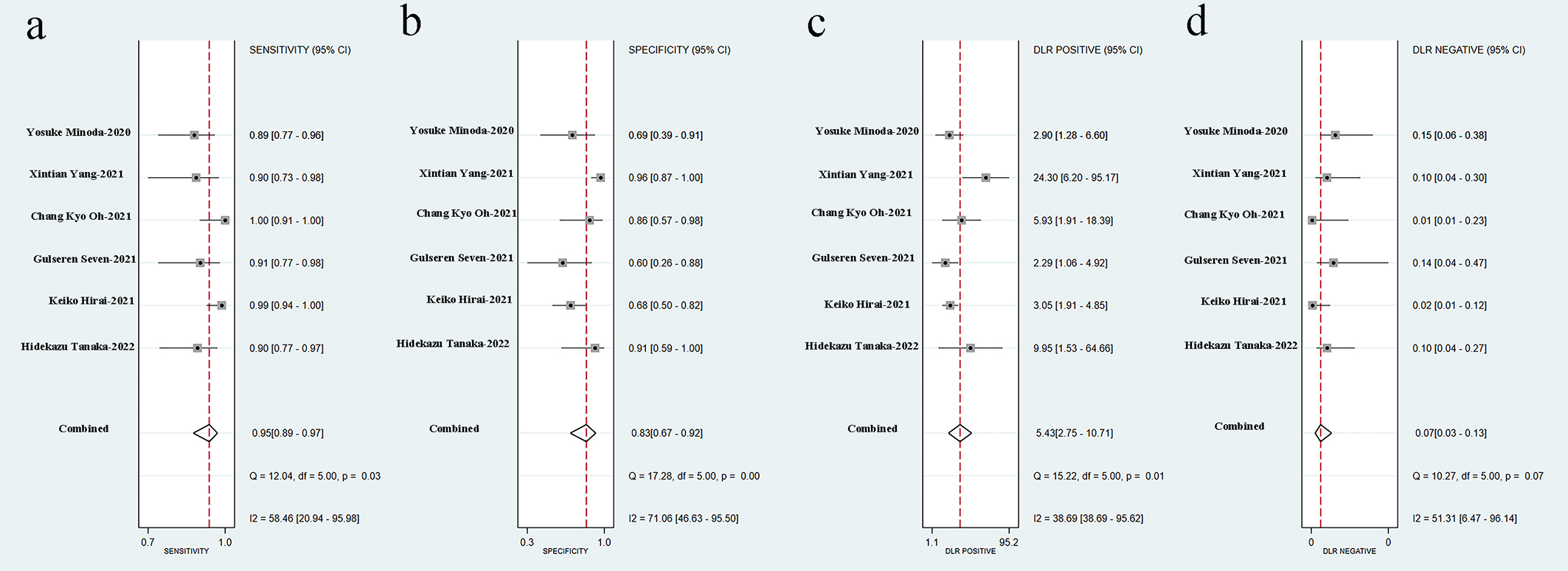

Supplement: Supplementary figure 8 — Sensitivity (A), specificity (B), positive likelihood ratio (C), negative likelihood ratio (D) of AI-assisted EUS of seven studies based on patients. [file Image_8.jpeg]
