# Supplementary material for: Application of artificial intelligence in the diagnosis of subepithelial lesions using endoscopic ultrasonography: a systematic review and meta-analysis
Source: Front Oncol. 2022 Aug 15;12:915481. doi: 10.3389/fonc.2022.915481 (PMC9420906; doi:10.3389/fonc.2022.915481)
Supplement: Supplementary Table 1 — The training, validation, and test datasets of included studies. [file Table_1.docx]

**Supplementary Table 1.** The training, validation, and test datasets of included studies.

| Study | Training set | | Validation set | | Test set* | |
| --- | --- | --- | --- | --- | --- | --- |
|  | GIST | Non-GIST | GIST | Non-GIST | GIST | Non-GIST |
| Yosuke Minoda-2020 | 112 | 61 | N/A | N/A | 47 | 13 |
| Yoon Ho Kim-2020 | 125 | 54 | N/A | N/A | 32 | 37 |
| Xintian Yang-2021 | 227 | 266 | 47 | 58 | 30/36** | 54/41** |
| Chang Kyo Oh-2021 | 85 | 29 | N/A | N/A | 40 | 14 |
| Keiko Hirai-2021 | 287 | 132 | 63 | 27 | 85 | 37 |
| Gulseren Seven-2021 | 74 | 26 | N/A | N/A | 35 | 10 |
| Hidekazu Tanaka-2022 | A 20-s CH-EUS video from each patient was divided into 0.1s intervals, yielding 200 images. | | | | 42 | 11 |
| Vien X. Nguyen-2010 | 50% of the data were used to train the neural network. | | | | 28 | 18 |

*If the study has only training and validation groups, consider the validation group as the test group.

**The former data is from retrospective diagnostic test set, the latter is from prospective diagnostic test set.

**Supplementary Table 2.** Types of SELs of included studies.

| Author | GIST | non- GIST | | | | | | Total non- GIST | Reference |
| --- | --- | --- | --- | --- | --- | --- | --- | --- | --- |
|  |  | Leiomyoma | Lipoma | Schwannoma | NET | Ectopic pancreas | Carcinoids |  |  |
| Vien X. Nguyen-2010 | 124*/28 | - | 106*/8 | - | - | - | 111*/10 | 217*/18 | (19) |
| Yosuke Minoda-2020 | 47 | 9 | - | 2 | - | 2 | - | 13 | (20) |
| Yoon Ho Kim-2020 | 106*/32 | 23 | - | 14 | - | - | - | 106*/37 | (21) |
| Xintian Yang-2021 | 30**/36 | 54**/41 | - | - | - | - | - | 54**/41 | (22) |
| Chang Kyo Oh-2021 | 40 | 14 | - | - | - | - | - | 14 | (23) |
| Keiko Hirai-2021 | 85 | 14 | - | 11 | 8 | 4 | - | 37 | (24) |
| Gulseren Seven-2021 | 35 | 10 | - | - | - | - | - | 10 | (25) |
| Hidekazu Tanaka-2022 | 42 | 11 | - | - | - | - | - | 11 | (26) |

*ROI, region of interest, not patient.

**Data of retrospective diagnostic test.
